# Longitudinal changes in health‐related quality of life after a breast cancer diagnosis in sub‐Saharan Africa: Evidence from the prospective ABC‐DO cohort

**DOI:** 10.1002/ijc.70350

**Published:** 2026-01-29

**Authors:** Shamsudeen Mohammed, Moses Galukande, Allen Naamala, Groesbeck Parham, Leeya Pinder, Angelica Anele, Shadrach Awa Offiah, Annelle Zietsman, Joachim Schüz, Valerie McCormack, Isabel dos‐Santos‐Silva

**Affiliations:** ^1^ Department of Non‐Communicable Diseases Epidemiology London School of Hygiene and Tropical Medicine London UK; ^2^ Department of Surgery, School of Medicine Makerere University Kampala Uganda; ^3^ UNC Zambia Lusaka Zambia; ^4^ Federal Medical Centre Owerri Nigeria; ^5^ Abia State University Teaching Hospital Aba Nigeria; ^6^ AB May Cancer Centre Windhoek Central Hospital Windhoek Namibia; ^7^ Environment and Lifestyle Epidemiology Branch International Agency for Research on Cancer Lyon France

**Keywords:** breast cancer, Global Health Status, health‐related quality of life, sub‐Saharan Africa

## Abstract

Breast cancer and its treatments impact on women's health‐related quality of life, but few studies have assessed these outcomes among survivors in sub‐Saharan Africa (SSA). We investigated longitudinal changes in Global Health Status (GHS) and their correlates within the African Breast Cancer‐Disparities in Outcomes cohort. Newly diagnosed women (≥18 years) across five SSA countries were recruited in 2014–2017. In four countries, follow‐up interviews were conducted 3‐monthly over 7 years using the European Organisation for Research and Treatment of Cancer Quality of Life 30‐item core questionnaire version 3. Multilevel mixed‐effects models identified correlates of GHS. Overall, 1358 women from Namibia, Nigeria, Uganda and Zambia were included, with a mean age of 50.6 (SD = 13.7) years. Median follow‐up time was 3 (IQR = 2–5) years, with a median of 18 GHS assessments per woman (IQR = 10–23). GHS was lowest in the first 6 months post‐diagnosis but improved gradually thereafter. GHS increased with increasing education (*p*‐value for linear trend [*p*_t_] < .0001), breast cancer awareness (adjusted‐odds ratio: 1.32; 95% confidence interval 1.22–1.42), surgical treatment (1.90; 1.53–2.37), family support (1.46; 1.24–1.71) and maintaining pre‐diagnosis employment (1.54; 1.41–1.68). In contrast, GHS decreased with increasing age at diagnosis (*p*_t_ <.0001), rural residence (0.76; 0.70–0.82), advanced tumour stage at diagnosis (*p*_t_ <.0001) and having non‐HIV comorbidities (0.74; 0.60–0.92). GHS was not affected by HIV status, but it was higher during COVID‐19 lockdowns than pre‐/post‐lockdown (1.27; 1.09–1.49). These patterns were similar at young and older ages, and in the short‐ and long‐term. The potentially modifiable factors associated with GHS are known to influence breast cancer survival in SSA. Addressing them could improve survivorship and survival from this cancer in the region.

## What's New?

Breast cancer is now the most common malignancy among women in sub‐Saharan Africa (SSA). Its impact on survivors’ quality of life and Global Health Status (GHS), however, remains inadequately studied in this setting. Here, using data from the African Breast Cancer‐Disparities in Outcomes (ABC‐DO) study, the authors examined GHS over time in SSA breast cancer survivors. Overall, GHS was lowest within the first 6 months post‐diagnosis and improved gradually thereafter. GHS was linked to potentially modifiable factors, including education level, breast cancer awareness and treatment. Addressing these sociodemographic and clinical GHS correlates could improve breast cancer survival in SSA.

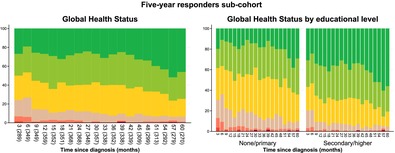

**Abbreviations**
ABC‐DO: African Breast Cancer‐Disparities in Outcomes
GBCI: Global Breast Cancer Initiative
GHS: Global Health Status
HRQoL: health‐related quality of life
SSA: sub‐Saharan Africa
WHO: World Health Organization

## 1 BACKGROUND

Breast cancer is the commonest cancer worldwide, with an estimated 2.3 million new cases diagnosed each year.^1^ Improvements in early detection and diagnosis coupled with more effective treatments have reduced considerably mortality from this disease since the late 1980s. Consequently, the number of survivors from the disease has increased, with an estimated 8.2 million women worldwide living, in 2022, with breast cancer diagnosed in the previous 5 years.^2^ Breast cancer survivors may suffer from a broad spectrum of adverse sequela associated with the cancer and its treatments, including pain, nausea, lymphedema, alterations in body image, sleep disturbances and anxiety, impacting their health‐related quality of life (HRQoL) and Global Health Status (GHS) both during and after treatment.^3^, ^4^, ^5^ The acute impact during active treatment can differ in nature and severity from long‐term effects.^6^

Breast cancer has recently overtaken cervical cancer to become the commonest female cancer in sub‐Saharan Africa (SSA).^2^ Although survival from breast cancer in SSA is poorer than in high‐income countries (HICs) (e.g., 5‐year survival: ~40% to 50% vs. 80%–90%, respectively),^7^, ^8^ with half of women being long‐term survivors, the number of survivors is sizeable and is projected to increase due to rises in breast cancer incidence coupled with expected improvements in survival following the recently launched World Health Organization (WHO) Global Breast Cancer Initiative (GBCI).^9^ Knowledge of GHS among survivors is required to ensure women receive appropriate care and support throughout their survivorship. However, few studies have examined GHS among breast cancer survivors in the region. A recent systematic review, which included studies published up to 2022,^10^ identified only nine breast cancer‐related QoL studies from SSA, most with cross‐sectional designs, small sample sizes and participation restricted to women undergoing specific cancer treatments or diagnosed at particular disease stages.

Although the HRQoL/GHS of breast cancer survivors in HICs has been extensively studied,^11^, ^12^ the findings cannot be generalised to survivors in SSA due to marked differences in socio‐cultural backgrounds, disease profiles, healthcare access and treatment options,^13^, ^14^, ^15^ including higher HIV prevalence, higher percentage of mastectomies, less supportive care and less access to endocrine therapy in SSA.

The African Breast Cancer‐Disparities in Outcomes (ABC‐DO) study,^7^, ^16^ a multicountry prospective breast cancer cohort in SSA, followed women newly diagnosed with the disease for over 7 years, with trimonthly collection of self‐reported HRQoL. Herein, we used data from this unique study to examine longitudinal changes in HRQoL among breast cancer survivors in SSA, and to identify sociodemographic and clinical correlates.

## 2 METHODS

### 2.1 Study design and participants

Details on the ABC‐DO study protocol have been published elsewhere.^7^, ^16^ Briefly, women aged ≥18 years newly diagnosed with breast cancer were recruited, regardless of ethnicity, residence and insurance status, from eight tertiary hospitals across five SSA countries (Namibia, Nigeria, South Africa, Uganda and Zambia) from September 2014 to December 2017. The participating hospitals were selected based on logistics and scientific criteria (i.e., pre‐existing or developing collaborations with the International Agency for Research on Cancer (IARC), capacity to recruit at least 100 newly diagnosed women with breast cancer annually, and inclusion of sites representing diverse populations and health systems). The participating hospitals included Windhoek Central Hospital (Namibia), Federal Medical Centre Owerri and Abia State University Teaching Hospital Aba (Nigeria), Uganda Cancer Institute and Mulago Hospital (Uganda), UTH/Cancer Diseases Hospital and Kabwe General Hospital (Zambia) and Chris Hani Baragwanath Academic Hospital (South Africa). Both the Namibian and South African hospitals provide structured access to multi‐modality treatment (surgery, chemotherapy, radiotherapy and hormonal therapy), but whilst the South African site serves a local population within a 50 km radius of Soweto, the Namibian hospital serves the entire country. In Nigeria, Uganda and Zambia, access to breast cancer care varies according to the availability and affordability of services at the time of treatment.^7^

Participants completed a face‐to‐face interviewer‐administered baseline questionnaire and were then followed up for over 7 years via trimonthly telephone interviews. To minimise losses to follow‐up, a customised ABC‐DO mHealth app was developed to produce up‐to‐date lists of women who were due to be contacted.^17^ The mHealth app also ensured collection of standardised high‐quality data by allowing immediate electronic capture, with in‐built data quality checks, of questionnaire data during interviews, as well as abstraction of standardised clinical data from hospital records.

In all, 2313 women with a suspicious breast cancer agreed to participate in the study (99% participation rate). For this analysis, we excluded 720 women from South Africa, where GHS was not captured. From the other four countries, we excluded 128 who had prevalent cancer or a benign tumour and 107 who died or were lost to follow‐up before the first GHS assessment, thus leaving 1358 women in the analysis.

### 2.2 Outcome

Our outcome of interest was GHS assessed during trimonthly telephone interviews to ABC‐DO participants from 2014–2017 to 2022/2023, using a modified version of the European Organisation for Research and Treatment of Cancer (EORTC) Quality of Life 30‐item core questionnaire version 3 (QLQ‐C30)^18^—a validated multidimensional cancer‐specific instrument.^19^, ^20^ A single question from the EORTC QLQ‐C30 ascertained women's GHS: ‘*How would you rate your overall health during the past week?*’ Responses were scored on a 7‐point Likert scale from 1 = ‘very poor’ to 7 = ‘excellent.’ The EORTC QLQ‐C30 is a well‐established instrument with proven validity and reliability in sub‐Saharan African populations.^21^, ^22^, ^23^, ^24^ The single GHS item has been shown to have a strong correlation with other components of the QLQ‐C30^25^, ^26^, ^27^ and offers a practical measure of overall HRQoL without the need for more detailed questions, particularly in longitudinal studies involving repeated assessments.

### 2.3 GHS correlates

Potential correlates of GHS considered herein included sociodemographic data collected at the baseline face‐to‐face interview, social support data collected at the baseline and 6‐month follow‐up interviews, and clinical data obtained at the baseline and follow‐up interviews coupled with abstraction from medical records, as detailed in Table 1. Sociodemographic data included participants' educational level, determined by asking their highest formal education: none, primary, secondary (high school) or higher (technical or university) education. Age at diagnosis was grouped into four categories: <40, 40–49, 50–59 and ≥60 years. Place of residence was based on responses to whether their area was rural, village, town or city. Responses were grouped into urban (town or city) and rural (rural or village). Data on ownership of assets (vehicle, refrigerator, landline phone, gas or electric stove, bed and home ownership) and access to utilities (indoor water, flush toilet and electricity) were used to generate country–ethnicity‐specific tertiles of socioeconomic position (low, medium and high). Breast cancer knowledge was assessed by asking women whether they thought breast cancer was potentially curable if diagnosed and treated early. Two social support factors, living with a partner (yes/no) and the number of children living with the woman, were collected at baseline. At 6‐month follow‐up, additional support factors were assessed: receiving help with household chores, self‐paying for medical expenses, whether they had changed jobs or stopped working due to their diagnosis, and whether they felt supported by family. Breast cancer stage at baseline/diagnosis was determined using the American Joint Committee on Cancer (AJCC) Tumour, Node, and Metastasis (TNM) staging system (shown in Table 1) and subsequently re‐categorised as stages 0/I/II, III and IV in the analysis, representing early, locally advanced and metastatic disease, respectively. Non‐HIV comorbidities include high blood pressure, heart disease, diabetes, anaemia, chronic obstructive pulmonary disease (COPD), asthma, hepatitis, tuberculosis, other infections and other cancers.

**TABLE 1:** Characteristics of the African Breast Cancer‐Disparities in Outcomes cohort eligible to participate in the present health‐related quality of life (HRQoL) analysis.

	Full cohort	5‐year responders sub‐cohort
	*n* (%)	*n* (%)
All women	1358 (100.0)	465 (34.2)
Age at diagnosis (years)		
<40	328 (24.2)	89 (19.1)
40–49	387 (28.5)	149 (32.0)
50–59	329 (24.2)	120 (25.8)
≥60	314 (23.1)	107 (23.0)
Mean (SD)	50.6 (13.7)	51.4 (13.0)
Country		
Namibia—non‐black	101 (7.4)	72 (15.5)
Namibia—Black	370 (27.2)	178 (38.3)
Nigeria	331 (24.4)	54 (11.6)
Uganda	389 (28.6)	121 (26.0)
Zambia	167 (12.3)	40 (8.6)
Residence		
Urban	732 (53.9)	272 (58.5)
Rural	626 (46.1)	193 (41.5)
Marital status		
Married	675 (49.7)	232 (49.9)
Not married	683 (50.3)	233 (50.1)
Socioeconomic position		
Low	562 (41.4)	169 (36.3)
Medium	471 (34.7)	132 (28.4)
High	325 (23.9)	164 (35.3)
Education level		
None	150 (11.0)	38 (8.2)
Primary	451 (33.2)	138 (29.7)
Secondary	457 (33.7)	162 (34.8)
Higher	300 (22.1)	127 (27.3)
Breast cancer can be cured		
No or don't know	607 (44.7)	137 (29.5)
Yes	751 (55.3)	328 (70.5)
How many children live with you		
0	239 (17.6)	86 (18.5)
1	230 (16.9)	80 (17.2)
2	208 (15.3)	85 (18.3)
3+	593 (43.7)	181 (38.9)
Missing	88 (6.5)	33 (7.1)
Live with other relatives		
No	689 (50.7)	249 (53.5)
Yes	582 (42.9)	184 (39.6)
Missing	87 (6.4)	32 (6.9)
Feel supported by family^a^		
No or don't know	81 (6.0)	15 (3.2)
Yes	1105 (81.3)	429 (92.3)
Missing	172 (12.7)	21 (4.5)
Help with chores^a^		
No	525 (38.6)	194 (41.7)
Yes	661 (48.7)	250 (53.8)
Missing	172 (12.7)	21 (4.5)
Self‐pay medical expenses^a^		
No	631 (46.4)	261 (56.1)
Yes	555 (40.9)	183 (39.4)
Missing	172 (12.7)	21 (4.5)
Move jobs or stop working after cancer diagnosis^a^		
No job before diagnosis	523 (38.5)	197 (42.4)
Moved jobs/stopped	148 (10.9)	49 (10.5)
Maintained job	515 (37.9)	198 (42.6)
Missing	172 (12.7)	21 (4.5)
HIV‐positive		
No	1232 (90.7)	419 (90.1)
Yes	126 (9.3)	46 (9.9)
Other comorbidities^b^		
No	671 (49.4)	228 (49.0)
Yes	687 (50.6)	237 (51.0)
Body mass index		
<18.5	70 (5.2)	21 (4.5)
18.5 to <25	512 (37.7)	151 (32.5)
25 to <30	405 (29.8)	149 (32.0)
≥30	319 (23.5)	128 (27.5)
Missing	52 (3.8)	16 (3.4)
Mean (SD)	26.6 (5.9)	27.4 (6.3)
Tumour stage at diagnosis		
0 (Non‐invasive)	10 (0.7)	4 (0.9)
I (T1 N0 M0, T0 N1 M0)	84 (6.2)	45 (9.7)
IIA (T0 N1 M0, T1 N1 M0, T2 N0 M0)	190 (14.0)	111 (23.9)
IIB (T2 N1 M0, T3 N0 M0)	235 (17.3)	115 (24.7)
IIIA (T0/T1/T2 N2 M0, T3 N1/N2 M0)	240 (17.7)	85 (18.3)
IIIB (T4 N0/N1/N2 M0)	311 (22.9)	54 (11.6)
IIIC (T1‐T4 N3 M0)	73 (5.4)	18 (3.9)
IV (T1‐T4 N1‐N3 M1)	146 (10.7)	17 (3.7)
Missing	69 (5.1)	16 (3.4)
Received surgery		
No	427 (31.4)	114 (24.5)
Yes	759 (55.9)	330 (71.0)
Missing	172 (12.7)	21 (4.5)
Received chemotherapy		
No	356 (26.2)	121 (26.0)
Yes	830 (61.1)	323 (69.5)
Missing	172 (12.7)	21 (4.5)
Received radiotherapy		
No	905 (66.6)	322 (69.3)
Yes	281 (20.7)	122 (26.2)
Missing	172 (12.7)	21 (4.5)
Received endocrine therapy		
No	797 (58.7)	271 (58.3)
Yes	389 (28.6)	173 (37.2)
Missing	172 (12.7)	21 (4.5)
Number of deaths since diagnosis		
0 to <6 months	37 (4.5)	‐
6 to <12 months	122 (14.8)	‐
12 to <24 months	211 (25.6)	‐
24 to <36 months	182 (22.1)	‐
36 to <60 months	170 (20.6)	‐
≥60 months	103 (12.5)	‐

Follow‐up and HRQoL assessments	Median (Interquartile Range (IQR))	Median (IQR)
Median (IQR) years of follow‐up	3 (2–5)	4 (2–5)
Median (IQR) number of HRQoL assessments, overall	18 (10–23)	21 (18–24)
Median (IQR) number of HRQoL assessments per year		
0–11 months (*n women* = 1303)	3 (2–3)	3 (2–3)
12–23 months (*n women* = 1073)	3 (3–4)	4 (3–4)
24–35 months (*n women* = 836)	4 (3–4)	4 (3–4)
35–47 months (*n women* = 683)	4 (3–4)	4 (3–4)
48–59 months (*n women* = 571)	3 (3–4)	3 (3–4)
60–71 months (*n women* = 454)	3 (3–4)	3 (3–4)
72–83 months (*n women* = 246)	4 (3–5)	4 (3–5)
Overall median (IQR) interval between consecutive follow‐ups (months)	3.2 (3.0–4.0)	3.2 (3.0–3.9)
Median (IQR) interval between consecutive follow‐ups (*n* women), by follow‐up year^c^		
0–11 months (*n women* = 1303)	3.0 (3.0–3.4)	3.0 (3.0–3.4)
12–23 months (*n women* = 1073)	3.1 (3.0–3.9)	3.1 (3.0–3.8)
24–35 months (*n women* = 836)	3.1 (3.0–4.0)	3.0 (3.0–3.7)
35–47 months (*n women* = 683)	3.2 (3.0–4.1)	3.1 (3.0–3.9)
48–59 months (*n women* = 571)	3.3 (3.0–4.3)	3.2 (3.0–4.0)
60–71 months (*n women* = 454)	3.3 (3.0–4.3)	3.3 (3.0–4.3)
72–83 months (*n women* = 246)	3.5 (3.0–4.4)	3.5 (3.0–4.4)

*Note*: Missing = not asked or missing for social support factors. HIV = Human Immunodeficiency Virus. SD = Standard Deviation.

^a^ Data on the following variables were collected at 6 months post‐diagnosis: feel supported by family, need help with home chores, self‐pay medical expenses and move jobs or stop working.

^b^ Other comorbidities include high blood pressure, heart disease, diabetes, anaemia, chronic obstructive pulmonary diseases (COPD), asthma, hepatitis, tuberculosis, other infections and other cancers.

^c^ *n* (i.e., numbers in the parenthesis) represent the number of women with at least one HRQoL assessment interview in the specified follow‐up period.

### 2.4 Statistical methods

To account for the hierarchical structure of the data, in which repeated GHS measures were nested within women and women nested within countries, three‐level random intercept mixed‐effects ordinal logistic regression models were used to assess associations between potential correlates and the GHS scores, with effect estimates reported as adjusted‐odds ratios (ORs), with 95% confidence intervals (95% CI). These models assume a common OR for any dichotomy of GHS (e.g., scores ≥2 vs. score 1 or scores ≥5 vs. scores <5) and can be interpreted as the odds ratio for better (higher) GHS. The proportional odds assumption was tested and showed no significant deviation from proportional odds. Separate models were fitted for sociodemographic, social support and clinical factors. The sociodemographic model was adjusted only for sociodemographic variables; the social support model for both sociodemographic and social support variables; and the clinical model for all three sets of variables (Figure S1).

All models were further adjusted for interviewer, country/ethnicity, age at diagnosis, time since diagnosis and country‐specific Coronavirus disease 2019 (COVID‐19) lockdown periods (as some GHS assessments were conducted during these periods). Variance inflation factor was used to assess multicollinearity. Analyses were conducted overall and stratified by age at cancer diagnosis (<50 vs. ≥50 years) and time since diagnosis (≤24 months to capture the acute effect in the active treatment period versus 25–84 months for long‐term effects).

To allow comparison with other studies and published guidelines,^28^ multilevel linear mixed‐effects models were also fitted after using a linear transformation to rescale the raw GHS scores to a 0–100 range. These models estimate effect sizes as adjusted mean differences (MD) in scores in absolute percentage points (p.p.), where a single unit increase on the Likert scale represents 17 p.p.

Given the high mortality rates among ABC‐DO participants, GHS measurements during follow‐up include data from women in their final months or years of life who were increasingly removed from the surviving group. To disentangle potential better‐prognosis responder effect, that is, apparent changes in GHS with time since diagnosis due to death, analyses were repeated among women who survived and completed at least one GHS assessment after their fifth follow‐up year (5‐year surviving responders sub‐cohort).

The percentage of participants with missing data was low in the full cohort (ranging from 5.1% to 12.7%) and even lower in the 5‐year surviving responders sub‐cohort (ranging from 3.4% to 7.1%; Table 1), with no missingness observed for sociodemographic variables. The variables with the highest missingness were the three social support variables, whose data were collected only at the 6‐month follow‐up, at a time when many women had already passed away or were too ill to participate (Table 1). As the overall proportion of missingness was low and largely attributable to study design rather than selective nonresponse, all models were complete case analyses.

Group‐based Trajectory Modelling (GBTM) was used to examine within‐woman changes in GHS and to identify distinct clusters of trajectories since diagnosis. Binary logistic regression was then used to identify participants' characteristics associated with trajectory group membership.

All analyses were performed using STATA v18.

## 3 RESULTS

### 3.1 Baseline characteristics of the participants

The study population comprised 1358 newly diagnosed breast cancer women, 471 from Namibia (370 Black; 101 non‐Black), 331 from Nigeria, 389 from Uganda and 167 from Zambia. The mean age at diagnosis was 50.6 (SD = 13.7) years, with 24.2% aged <40 years (Table 1). Overall, 41.4% of the participants had low socioeconomic positions, 11.0% had no formal education, 53.9% lived in urban areas, 9.3% were known to be HIV‐positive and 50.6% had other non‐HIV comorbidities. In all, 10.7% were diagnosed with stage IV disease, 55.9% received surgical treatment, 61.1% chemotherapy, 20.7% radiotherapy and 28.6% hormone (endocrine) therapy. Median follow‐up time since diagnosis was 3 years (IQR 2–5 years). Women completed a median of 18 (IQR = 10–23) GHS assessments, with 465 (34.2%) women having completed at least one quality of life (QOL) measurement after 5 years of follow‐up. Relative to the full cohort, these 5‐year surviving responders had higher socioeconomic status and were more likely to have been diagnosed with early‐stage disease and to have received treatment (Table 1). The median interval between consecutive assessments was, as per the study protocol, ~3 months throughout the follow‐up. In the first year following diagnosis, 19.3% of the women died.

### 3.2 Longitudinal changes in GHS

**FIGURE 1:**
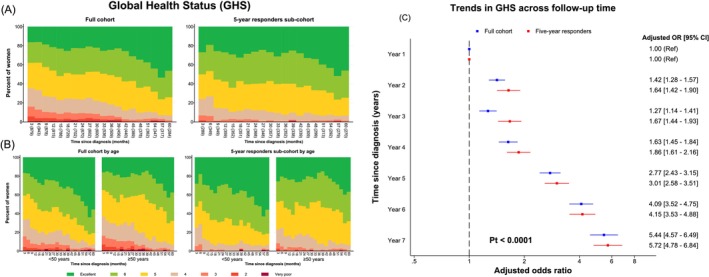
Distribution and trends in Global Health Status (GHS) in the full cohort of women diagnosed with breast cancer and in the 5‐year responders sub‐cohort, (A) overall, (B) by age at diagnosis and (C) follow‐up time. Time since diagnosis was rounded to the nearest 3‐month interval as follow‐up times were not evenly spaced. GHS measurements during COVID‐19 pandemic lockdowns were excluded. For (A), numbers in parentheses represent the number of women who completed each follow‐up GHS assessment. The number of women increased between the 3‐month and subsequent assessments in the first year of follow‐up because some joined the African Breast Cancer‐Disparities in Outcomes study after their diagnosis was confirmed but before the start of any curative treatment. For (C), odds ratios were adjusted for all sociodemographic factors, interviewer and COVID‐19 lockdowns. *p*_t_ = *p*‐value for linear trend.

In the first 6 months post‐diagnosis, 64.6% of participants rated their GHS as good, very good or excellent (mean scores of 4.9 at 3 months and 5.0 at 6 months), hereafter termed good+ GHS (scores of 5–7; Figure 1A). This percentage increased to 73.7% by 12 months, remained stable thereafter, and reached 81.5% at 3 years and 93.0% at the end of the fifth post‐diagnosis year. There were, however, variations by country‐ethnicity, with women in Nigeria, Uganda and Zambia reporting poorer GHS than elsewhere (Figure S2). A similar all country‐ethnicity pattern was observed among the 5‐year responders, except that the percentage rating their GHS as good+ at 3 months post‐diagnosis was higher (75.8%) than in the full cohort and increased more markedly afterwards (Figure 1A). Consequently, the OR of better GHS between 1 and 5 years since diagnosis doubled in both the full cohort and among 5‐year responders (Figure 1C) corresponding, respectively, to a MD of +4.88 (95% CI 3.80–5.96) and +8.74 (7.64–9.83) p.p. (Figure S3).

### 3.3 GHS correlates

**FIGURE 2:**
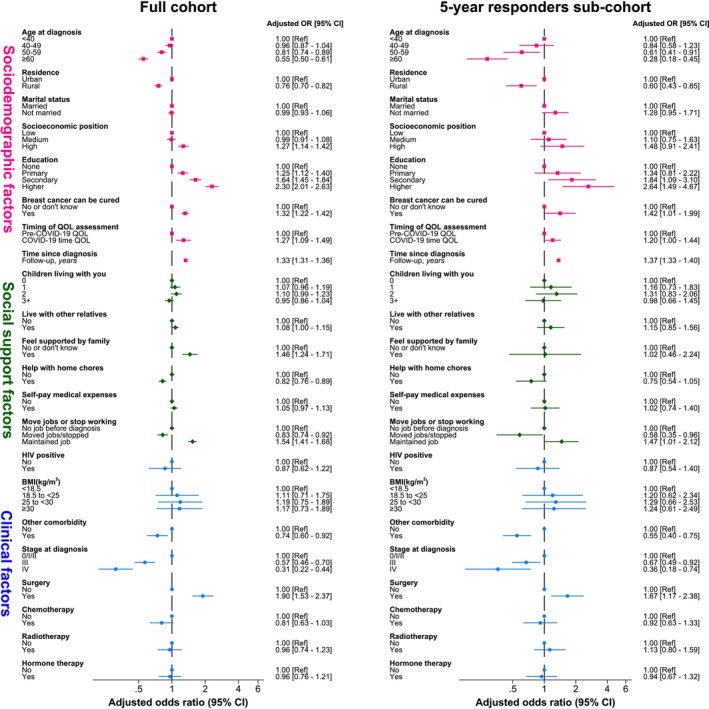
Correlates of Global Health Status (GHS) in the full cohort of women diagnosed with breast cancer and in the 5‐year responders sub‐cohort. *p*‐Value for a linear trend (*p*_t_) in the odds of better GHS for ordered categorical correlates in the full cohort: *p*_t_ <.0001 for age at diagnosis, socioeconomic position, education and stage at diagnosis; *p*_t_ <.05 for number of children; *p*_t_ >.05 for body mass index (BMI). *p*_t_ in the 5‐year responder sub‐cohort: *p*_t_ <.01 for tumour stage at diagnosis; *p*_t_ >.05 for age at diagnosis, socioeconomic position, education, number of children and BMI. CI, confidence interval.

Overall, that is, pooling together data from all completed GHS assessments, the ORs of better GHS decreased with older age at cancer diagnosis and rural residence, but increased with increasing education, high socioeconomic position and breast cancer knowledge (Figure 2). No association was seen with marital status. The education‐GHS association was particularly strong, with women with higher education being 2.30 (95% CI 2.01–2.63) times more likely to report better GHS than those with no formal education (*p*‐value for linear trend (*p*_t_) < .0001), corresponding to an MD of +8.18 (95% CI 4.71–11.65) p.p. (Figure S4). Noteworthy, women reported better GHS scores during COVID‐19 lockdown compared to pre/post‐lockdown period (OR = 1.27; 95% CI 1.09–1.49). The ORs of better GHS scores were higher among women who felt supported by their families, but lower among those who needed help with home chores (Figure 2). Relative to women who held no job before the diagnosis, the OR of better GHS was higher among those who were able to keep their pre‐diagnostic job but lower among those who moved jobs or stopped working. Further adjustment for clinical variables did not change appreciably the magnitude of the GHS associations with socioeconomic and social support variables, with, for instance, the ORs of better GHS among women with higher education versus those with no formal education being 2.30 (95% CI 2.01–2.63; Figure 2) and 2.03 (1.34–3.07; not shown), respectively, before and after further adjustment for clinical variables.

GHS was strongly influenced by tumour stage at diagnosis, with women diagnosed at stages III and IV having, respectively, a 43% (OR = 0.57; 95% CI 0.46–0.70) and a 69% (OR = 0.31; 0.22–0.44) reduced odds of reporting a better GHS than those diagnosed at stage 0/I/II (Figure 2). The OR of better GHS was higher among women who underwent surgery, but lower among those who received chemotherapy, whilst radiotherapy and endocrine therapy had no overall effect. Being HIV‐positive at the time of the cancer diagnosis did not affect GHS, but having other comorbidities was associated with lower odds of better GHS (OR = 0.74, 95% CI 0.60–0.92).

Similar GHS associations with sociodemographic, social support and clinical variables were present among 5‐year responders, but with less precise OR estimates, that is, with wider CIs, due to smaller numbers (Figure 2).

### 3.4 GHS by age at cancer diagnosis

**FIGURE 3:**
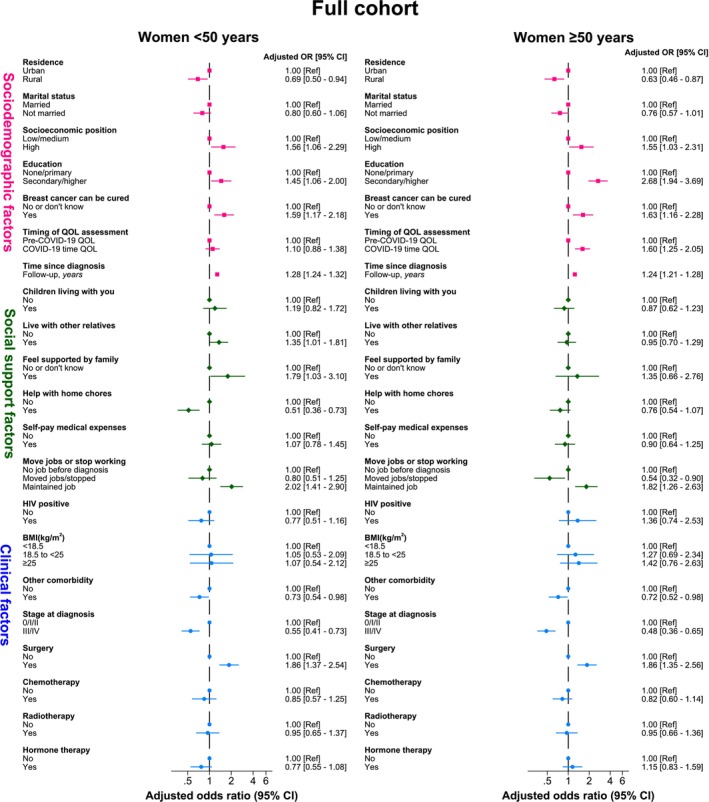
Correlates of Global Health Status, by age at diagnosis, in the full cohort of women diagnosed with breast cancer. CI, confidence interval.

The percentage of participants who rated their GHS as good+ increased more markedly in the first 3 years of follow‐up among those aged <50 years at cancer diagnosis than among those aged ≥50 years in both the full cohort and the 5‐year responders sub‐cohort (Figure 1B). Consequently, the OR for better GHS decreased progressively with increasing age (*p*_t_ <.0001) throughout follow‐up (Figure 2), with the magnitude of these estimates in both the full cohort and in the 5‐year responders sub‐cohort changing little with further adjustment for clinical variables (data not shown). However, similar correlates of GHS to those identified at all‐ages were seen at ages <50 and ≥50 years both in the full cohort (Figure 3) and in the 5‐year responders sub‐cohort (Figure S5), except that the higher odds of reporting better GHS during the COVID‐19 pandemic was only seen among women aged ≥50 years.

### 3.5 Correlates of short‐ and long‐term GHS

**FIGURE 4:**
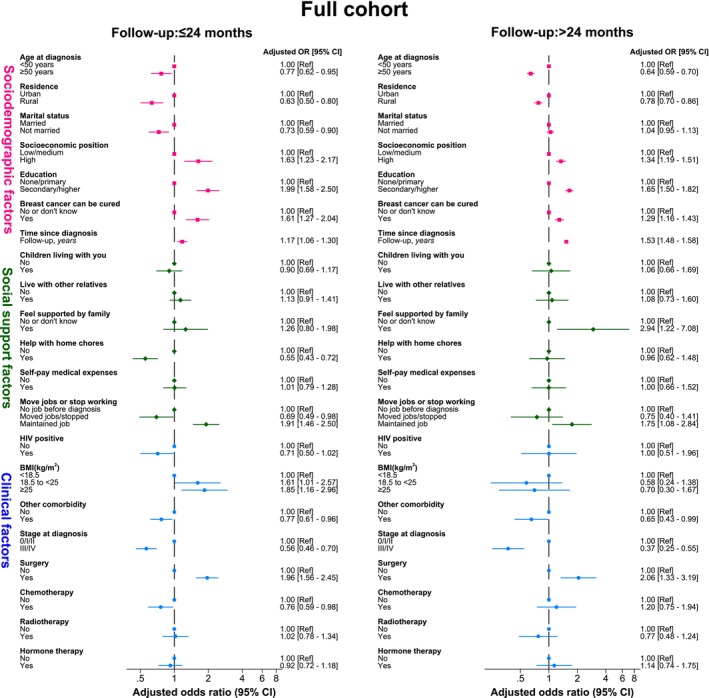
Correlates of Global Health Status, by time since diagnosis, among the full cohort of women diagnosed with breast cancer. CI, confidence interval.

Similar sociodemographic, social support and clinical associations with GHS were observed in the short‐ (i.e., within the first 24 months post‐diagnosis) and long‐term, with only a few exceptions (Figure 4). Being unmarried, needing help with home chores, having to move jobs/stop working, receiving chemotherapy and having comorbidities were all inversely associated with the odds of reporting a higher GHS score in the short‐term, but albeit similar (or null) inverse associations were seen in the long‐term; most factors were not statistically significant. Similarly, a positive trend in GHS with increasing BMI was observed in the short‐term, but not in the long‐term (*p*‐value for interaction = .034). Analyses restricted to 5‐year responders (Figure S6) revealed similar associations but with wider CIs around the OR estimates.

### 3.6 Within‐woman changes in GHS

**FIGURE 5:**
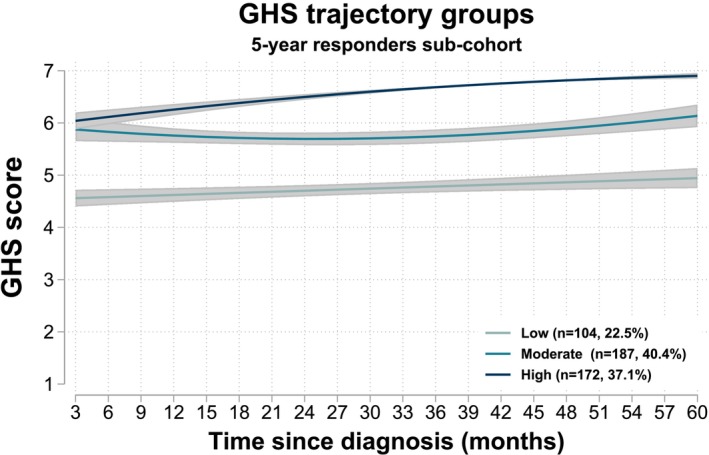
Global Health Status (GHS) trajectory groups identified by Group‐based Trajectory Modelling among the 5‐year responders sub‐cohort. Shaded areas around solid lines represent 95% confidence intervals. The number of trajectory groups was determined on the basis of the Akaike Information Criterion (AIC), Bayesian Information Criterion (BIC), odds of correct classification (OCC), entropy, average posterior probability of class assignment (AvPPA) and percentage distribution, with lower AIC and BIC values indicating better model fit, and higher entropy, OCC and AvPPA values indicating better classification quality.

After evaluating model fit indices and trajectory patterns, three GHS trajectory groups were identified (Figure 5) and labelled low (*n* = 104, 22.5%), moderate (*n* = 187, 40.4%) and high (*n* = 172, 37.1%). The average GHS score for women in the low group steadily increased from 4.6 at 3 months to 4.9 at 60 months, whilst those for women in the moderate group declined from 5.9 at 3 months to 5.7 at 36 months, increasing slightly thereafter. Women in the high group showed the most improvement, rising from an average score of 6.0 at 3 months to an average score of 6.9 at 60 months. Women were more likely to be in the high trajectory group if they were aged <50 years at diagnosis, lived in urban areas, had secondary/higher education, were breast cancer aware, kept their pre‐diagnostic job and had no non‐HIV comorbidities (Figure S7).

## 4 DISCUSSION

### 4.1 Main findings

Herein, we investigated GHS patterns and correlates within the large prospective ABC‐DO cohort of newly diagnosed breast cancer women in SSA who were followed up for up to 7 years. The findings showed improvement in GHS with increasing time since diagnosis both in the full cohort and in the 5‐year responders sub‐cohort. Several sociodemographic, social support and clinical correlates of GHS were identified, with their impact being similar in younger and older women at diagnosis, and in the short‐ (post‐diagnostic treatment period) and long‐term. GHS was positively associated with educational level, socioeconomic position, urban residence and awareness that breast cancer is potentially curable, but inversely associated with age at cancer diagnosis. Women who felt supported by their families, and those who were able to maintain their pre‐diagnosis job, were also more likely to report better GHS. In contrast, needing help with household chores 6 months post‐diagnosis was negatively associated with GHS, possibly reflecting pre‐existing poor health. Advanced breast cancer stage at diagnosis and presence of non‐HIV comorbidities were both associated with worse GHS, whilst receiving surgical treatment was associated with better GHS. GHS did not differ by HIV status at diagnosis, but surprisingly, women reported better GHS during COVID‐19 lockdowns than in the pre‐ or post‐lockdown periods.

### 4.2 Comparisons with other studies

Our cohort scored higher on the single‐item HRQoL/GHS scale than the EORTC QLQ‐C30 international reference values.^18^ The scores were also higher than those reported in published meta‐analyses^12^, ^29^ (Table S1). Consistent with our findings, prior non‐SSA longitudinal studies^30^, ^31^ have shown that the HRQoL of women with breast cancer improves over time. The improvement in GHS suggests that many SSA women experience a recovery in perceived health status after the initial impact of diagnosis and treatment, possibly reflecting physical recovery, psychological adaptation or an increase in coping capacity.

Studies that assessed the impact of age on HRQoL/GHS have reported inconsistent results,^29^, ^32^, ^33^ albeit a recent global meta‐analysis^12^ reported better HRQoL with advancing age at diagnosis. In addition, many previous studies^29^, ^33^, ^34^ have reported better HRQoL in women with education and knowledge about breast cancer, in line with our findings. The positive effects of maintaining pre‐diagnosis employment, having higher socioeconomic status, and living in urban areas on HRQoL are well‐documented.^33^, ^34^, ^35^ These factors often contribute to improved healthcare access and the financial ability to afford treatment, a critical issue in most SSA settings with no universal healthcare. Maintaining employment may also provide a sense of normalcy through routine and familiar social interactions^35^ and could shift focus from the illness. This may also explain why women with supportive families reported better GHS.

In line with our findings, previous studies^12^, ^32^, ^33^ have shown that both advanced tumour stage at diagnosis and presence of non‐HIV comorbidities are associated with poorer HRQoL. HIV‐positive women experience poorer survival after a breast cancer diagnosis than their HIV‐negative counterparts^36^ but, in line with a previous study from SSA,^37^ HIV status did not affect GHS in our cohort perhaps because most HIV‐positive women (78%) were on antiretroviral therapy (ART),^36^ which has been shown to reduce disparities in HRQoL between HIV‐positive and HIV‐negative cancer‐unaffected individuals.^38^ Noteworthy, however, women (≥50 years) reported better GHS during the COVID‐19 lockdowns, a finding that contrasts with those from previous studies.^39^, ^40^ The reasons for this are unclear, but may partly reflect the fact that previous studies had focused on women who were still undergoing cancer treatment, whilst most ABC‐DO women had already completed treatment by the time the pandemic reached SSA. The type of cancer treatment received is also known to affect GHS. Women who undergo mastectomy without reconstruction often report lower HRQoL than those with breast‐conserving surgery or reconstruction,^41^, ^42^ although some studies have reported no HRQoL difference.^43^, ^44^ However, the few studies comparing surgical intervention to no surgery, as in our cohort, have generally reported no significant association with HRQoL.^45^, ^46^ In our study, surgery was associated with better GHS despite most women having undergone radical mastectomy without reconstruction.

### 4.3 Strengths and limitations

Strengths of the ABC‐DO study include its large sample size, long‐term follow‐up (up to 7 years), minimal losses to follow‐up (16% at 7 years),^47^ wealth of data on sociodemographic, social support and clinical factors and regular trimonthly GHS assessments. To our knowledge, ABC‐DO is the first longitudinal study in SSA to assess GHS over a long follow‐up period. The prospective design minimised the potential for the observed associations between baseline factors and GHS scores to have been affected by reverse causality, an issue which affects the interpretation of findings from the more widely‐used cross‐sectional design. Analyses were conducted in the full cohort and also among the 5‐year responders sub‐cohort to address potential better‐prognosis responder effects. However, in contrast to what is seen in studies in HICs in which selective death of poor‐prognosis patients leads to a more marked improvement in GHS with time since diagnosis among the full cohort than among the survivor sub‐cohort, the opposite was observed in our study (Figure 1) because 67% (552/825) of the observed deaths occurred within the first 3 years of the breast cancer diagnosis (19.3% in Year 1, 25.6% in Year 2, 22.1% in Year 3; Table 1), reflecting the poor overall survival from the disease in SSA.^7^

Limitations of the study included a possible lack of representativeness as participants were recruited from public tertiary hospitals located predominantly in capital cities and, hence, may be unrepresentative of all breast cancer patients in the region. Exclusion of the South African participants might also have introduced geographical bias, limiting the generalisability of our findings to the broader sub‐Saharan African context. Nonetheless, the countries retained in the analysis still represented diverse settings within SSA, patient profiles and health system capacities, thereby improving the relevance of our findings to similar contexts within the region. The use of a single item from a single scale to measure GHS, albeit it has been used by others,^25^ may limit comparisons with multi‐item studies. Reliance on self‐reported measures of GHS might have introduced measurement errors, and if these were differential, also response bias. Adjustment was made for many sociodemographic, clinical and social support variables; residual confounding by errors in the measurement of these variables (e.g., available data on treatment was rather crude) as well as by unmeasured factors (e.g., psychosocial support) cannot be ruled out and may have affected the magnitude of the observed effect estimates. Finally, although the magnitude of the odds of reporting a better GHS with time since diagnosis was considerable, the magnitude of the corresponding MD in absolute p.p. was, as seen in other studies,^28^, ^48^ rather small, reflecting perhaps response shift bias caused by psychological adaptation of patients to their changing health.^49^

### 4.4 Implications of the findings

Our findings showed that GHS of breast cancer patients in SSA is comparable to those in HICs^30^, ^31^ despite marked differences in their socio‐cultural backgrounds, disease profiles, healthcare access, treatment options and support networks and the much poorer survival from the disease in SSA. The potentially modifiable correlates of GHS identified in this study are also known to affect survival from the disease in the region and, thus, addressing them will bring about improvements in both survival and survivorship. Implementing the GBCI pillars, which aim to reduce breast cancer mortality, will also likely improve GHS. Interventions to evaluate the impact of breast cancer awareness on GHS are recommended as they should encourage early presentation. Health systems improvements could facilitate early diagnosis by streamlining referral pathways, given that over 60% of delays occur during the diagnostic journey.^50^ Our findings also underscore the need for early supportive care interventions, especially in the first year post‐diagnosis when GHS is lowest. Furthermore, initiatives to improve healthcare access in rural areas and address socioeconomic disparities through measures such as government subsidies could ease the financial burden of breast cancer diagnosis and treatment and, consequently, the socioeconomic disparities in GHS. In conclusion, our study provides a deeper understanding of breast cancer survivorship in SSA, identifying important areas for interventions to improve GHS.

## AUTHOR CONTRIBUTIONS

**Shamsudeen Mohammed:** Writing – original draft; writing – review and editing; formal analysis; visualization; methodology; validation. **Moses Galukande:** Writing – review and editing; investigation; methodology. **Allen Naamala:** Investigation; writing – review and editing; methodology. **Groesbeck Parham:** Investigation; writing – review and editing; methodology. **Leeya Pinder:** Investigation; writing – review and editing; methodology. **Angelica Anele:** Investigation; writing – review and editing; methodology. **Shadrach Awa Offiah:** Investigation; methodology; writing – review and editing. **Annelle Zietsman:** Investigation; methodology; writing – review and editing. **Joachim Schüz:** Investigation; methodology; writing – review and editing; supervision. **Valerie McCormack:** Investigation; methodology; writing – review and editing; conceptualization; funding acquisition; writing – original draft; visualization; formal analysis; project administration; supervision; data curation; resources; validation. **Isabel dos‐Santos‐Silva:** Conceptualization; investigation; funding acquisition; writing – original draft; methodology; visualization; writing – review and editing; formal analysis; project administration; data curation; supervision; resources; validation.

## FUNDING INFORMATION

The African Breast Cancer‐Disparities in Outcomes study was funded by the National Cancer Institute (1R01CA244559), Susan G Komen (grants IIR 13264158, GSP18IARC001 and GSP19IARC001) and the International Agency for Research on Cancer.

## CONFLICT OF INTEREST STATEMENT

All authors declare no conflicts of interest.

## ETHICS STATEMENT

The African Breast Cancer‐Disparities in Outcomes study was approved by the ethics committees of all involved institutions. All participants provided written informed consent or, if illiterate, a fingerprint.

## Supporting information


**Data S1.** Supporting Information.

**Table S1.** Comparison of transformed EORTC QLQ‐C30 HRQoL scores.

## Data Availability

Deidentified data and further information that supports the findings of this study are available from the corresponding authors upon request.
