# Supplementary material for: Longitudinal changes in health‐related quality of life after a breast cancer diagnosis in sub‐Saharan Africa: Evidence from the prospective ABC‐DO cohort
Source: Int J Cancer. 2026 Jan 29;159(1):78–91. doi: 10.1002/ijc.70350 (PMC13139992; doi:10.1002/ijc.70350)
Supplement: Supplementary file 1 — Data S1. Supporting Information. [file IJC-159-78-s001.pdf]

Longitudinal changes in health-related quality of life after a breast cancer diagnosis in sub-Saharan Africa: evidence from the prospective ABC-DO cohort

Shamsudeen Mohammed, Moses Galukande, Allen Naamala, Groesbeck Parham, Leeya Pinder, Angelica Anele, Shadrach Awa Offiah, Annelle Zietsman, Joachim Schüz, Valerie McCormack, Isabel dos-Santos-Silva

Table of Contents

Supplementary Figure 1	Directed acyclic graph illustrating the hypothesised relationships among (1) sociodemographic, (2) social support, and (3) clinical factors in relation to Global Health Status (GHS).
Supplementary Figure 2	. Distribution of the GHS in the full cohort of women diagnosed with breast cancer and in the 5-year responders sub-cohort, by country.
Supplementary Figure 3	Trends in GHS by time since diagnosis among the full cohort of women diagnosed with breast cancer and among the 5-year responders sub-cohort.
Supplementary Figure 4	Correlates of GHS among the full cohort of women diagnosed with breast cancer and among the five-year responders sub-cohort.
Supplementary Figure 5	Correlates of HRQoL, by age at diagnosis, in the full cohort of women diagnosed with breast cancer and in the 5-year responders sub-cohort.
Supplementary Figure 6	Correlates of HRQoL, by time since diagnosis, in the 5-year responders subcohort of women diagnosed with breast cancer.
Supplementary Figure 7	Binary logistic regression analysis of the factors associated with HRQoL trajectory group membership in the 5-year responders sub-cohort of women diagnosed with breast cancer.

1. Sociodemographic model

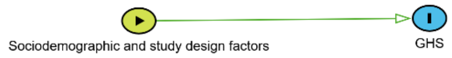

2. Social support model

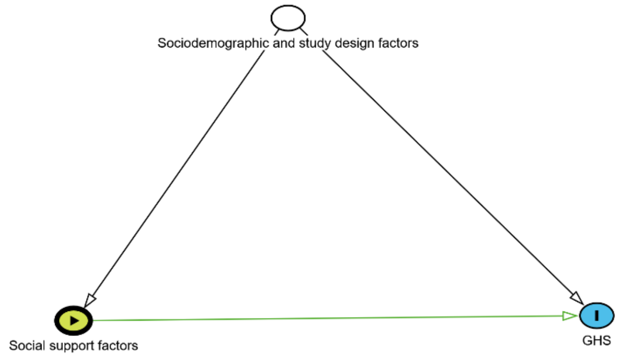

3. Clinical model

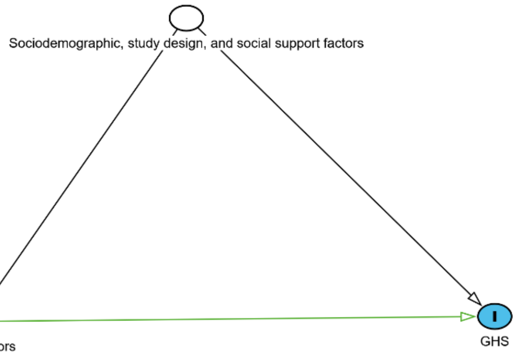

Sociodemographic and study design factors
Age at diagnosis
Residence
Marital status
Sociodemographic position
Education
Breast cancer knowledge
Time since diagnosis
Timing of QOL assessment (COVID-19 lockdown period)
Interviewer
Country/ethnicity

Social support factors
Children living with you
Live with other relatives
Feel supported by family
Help with home chores
Self-pay medical expenses
Move jobs or stop working

Clinical factors
Surgery
Chemotherapy
Radiotherapy
Stage at diagnosis
HIV positive
BMI
Other comorbidity

Supplementary Figure 1. Directed acyclic graph illustrating the hypothesised relationships among (1) sociodemographic, (2) social support, and (3) clinical factors in relation to Global Health Status (GHS).

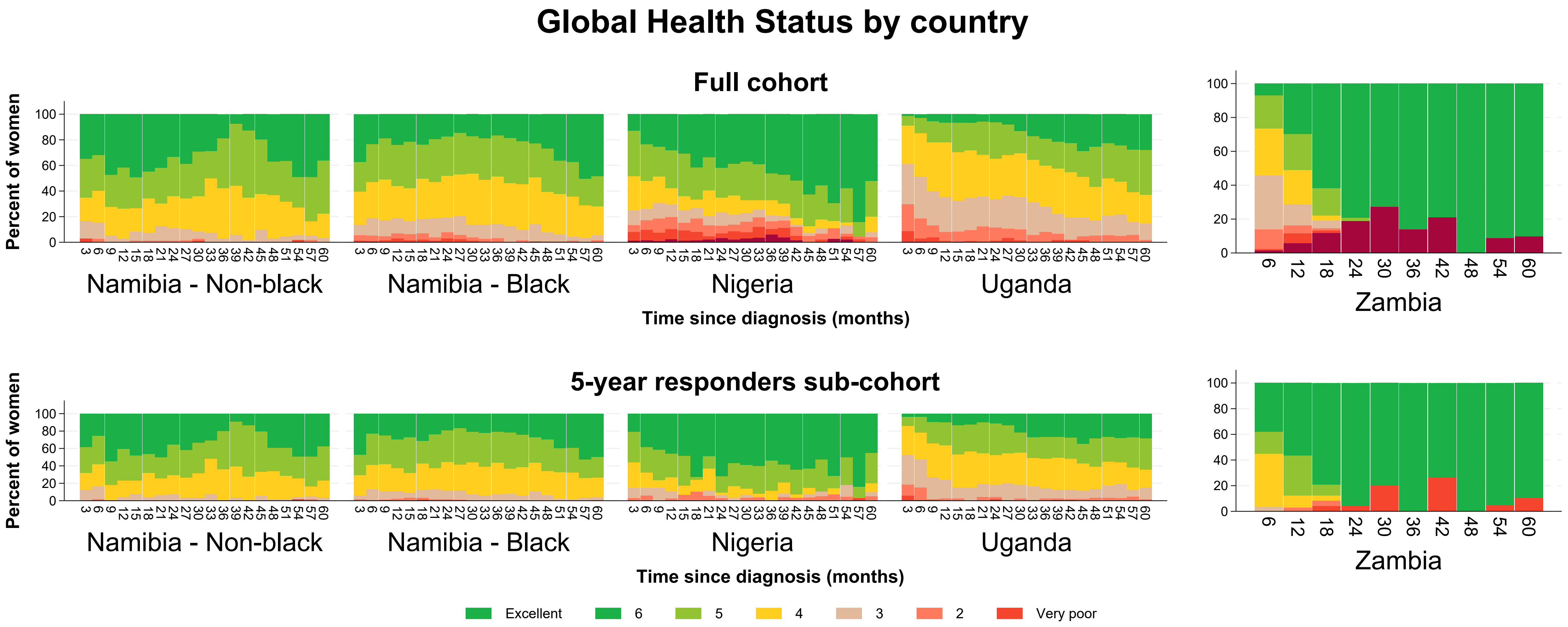

Supplementary Figure 2. Distribution of the GHS in the full cohort of women diagnosed with breast cancer and in the 5-year responders sub-cohort, by country.

Time since the first follow-up was rounded to the nearest 3-month interval as follow-up times were not evenly spaced. GHS assessments during each country's COVID-19 pandemic lockdown were excluded. Six-monthly patterns are shown for Zambia due to small number of observations at the trimonthly follow-ups.

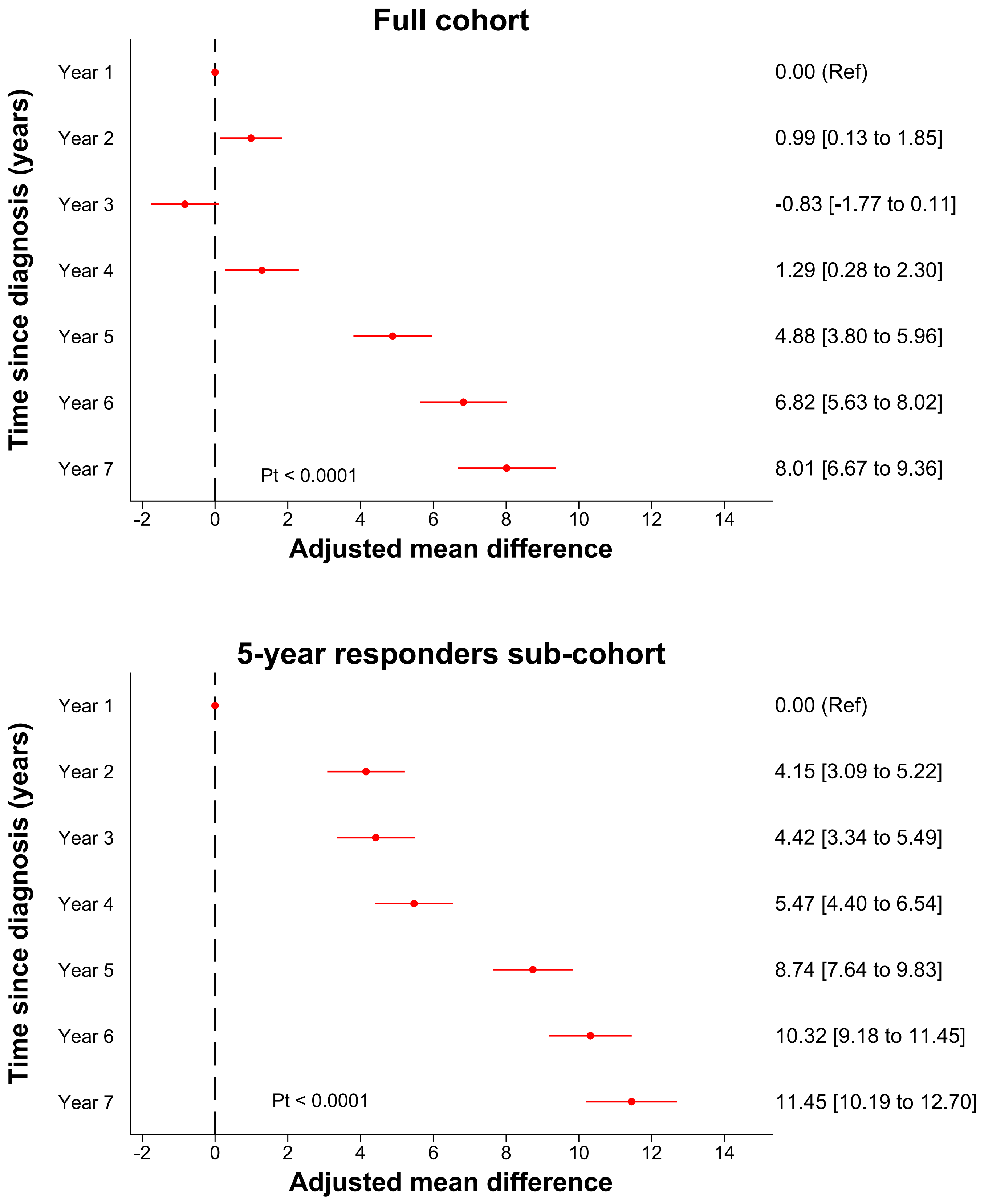

Supplementary Figure 3. Trends in GHS by time since diagnosis among the full cohort of women diagnosed with breast cancer and among the 5-year responders sub-cohort.

GHS scores were linearly transformed according to the EORTC guidelines to range from 0 to 100. Estimates are mean differences in transformed GHS scores adjusted for sociodemographic factors, months since diagnosis, age at each follow-up, interviewer, and COVID-19 pandemic. Pt = P-value for linear trend.

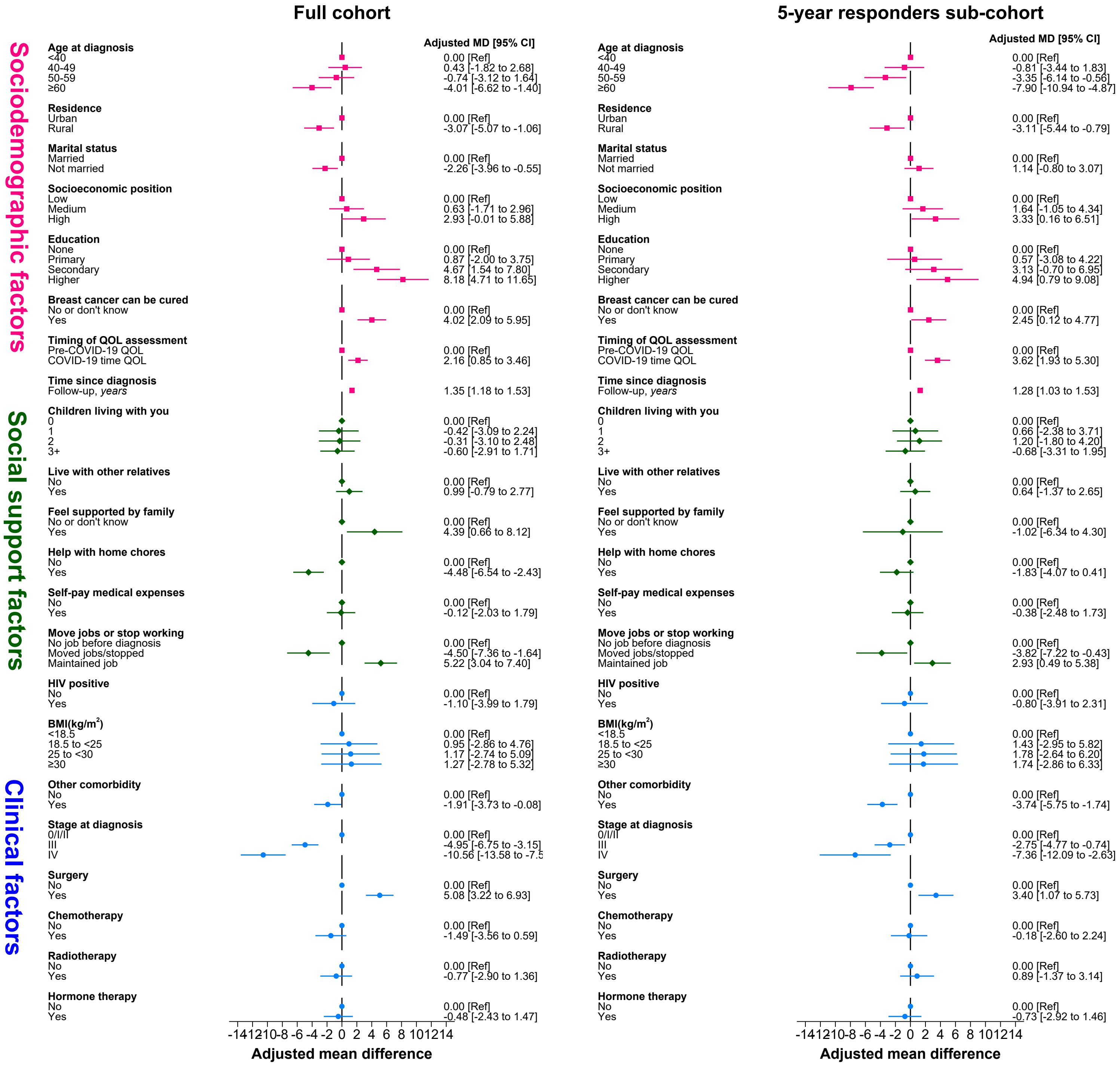

Supplementary Figure 4. Correlates of GHS among the full cohort of women diagnosed with breast cancer and among the five-year responders sub-cohort.

GHS scores were linearly transformed following EORTC guideline to range from 0 to 100. P-value for a linear trend (Pt) in the odds of better GHS for ordered categorical correlates in the full cohort: $p<0.0001$ for education and stage at diagnosis; $p<0.01$ for age at diagnosis; $p>0.05$ for socioeconomic position, number of children, and BMI. Pt in the five-year responder sub-cohort: $p<0.01$ for age at diagnosis, education, and stage at diagnosis; $p>0.05$ for socioeconomic position, number of children, and BMI.

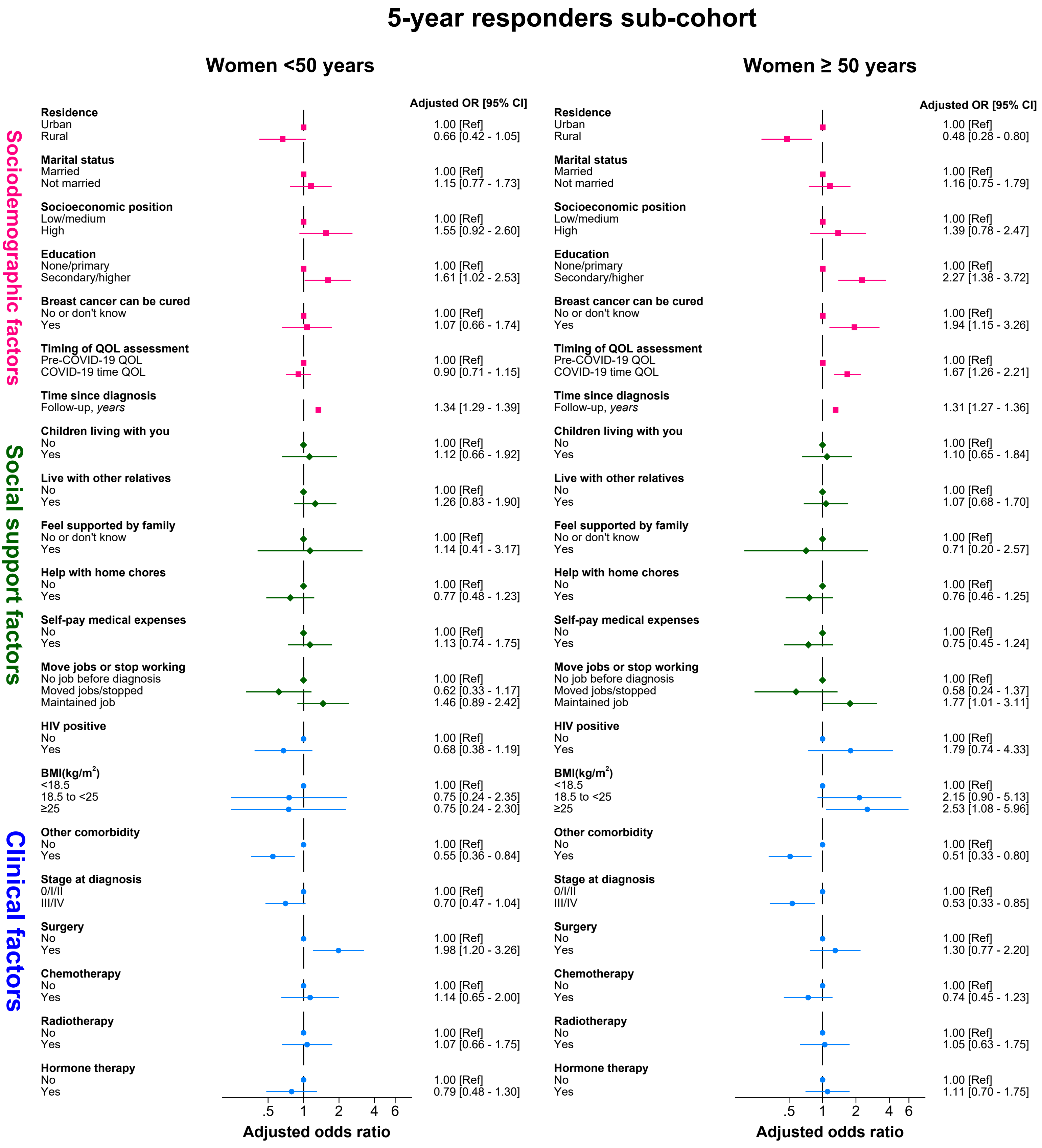

Supplementary Figure 5. Correlates of HRQoL, by age at diagnosis, in the full cohort of women diagnosed with breast cancer and in the 5-year responders sub-cohort.

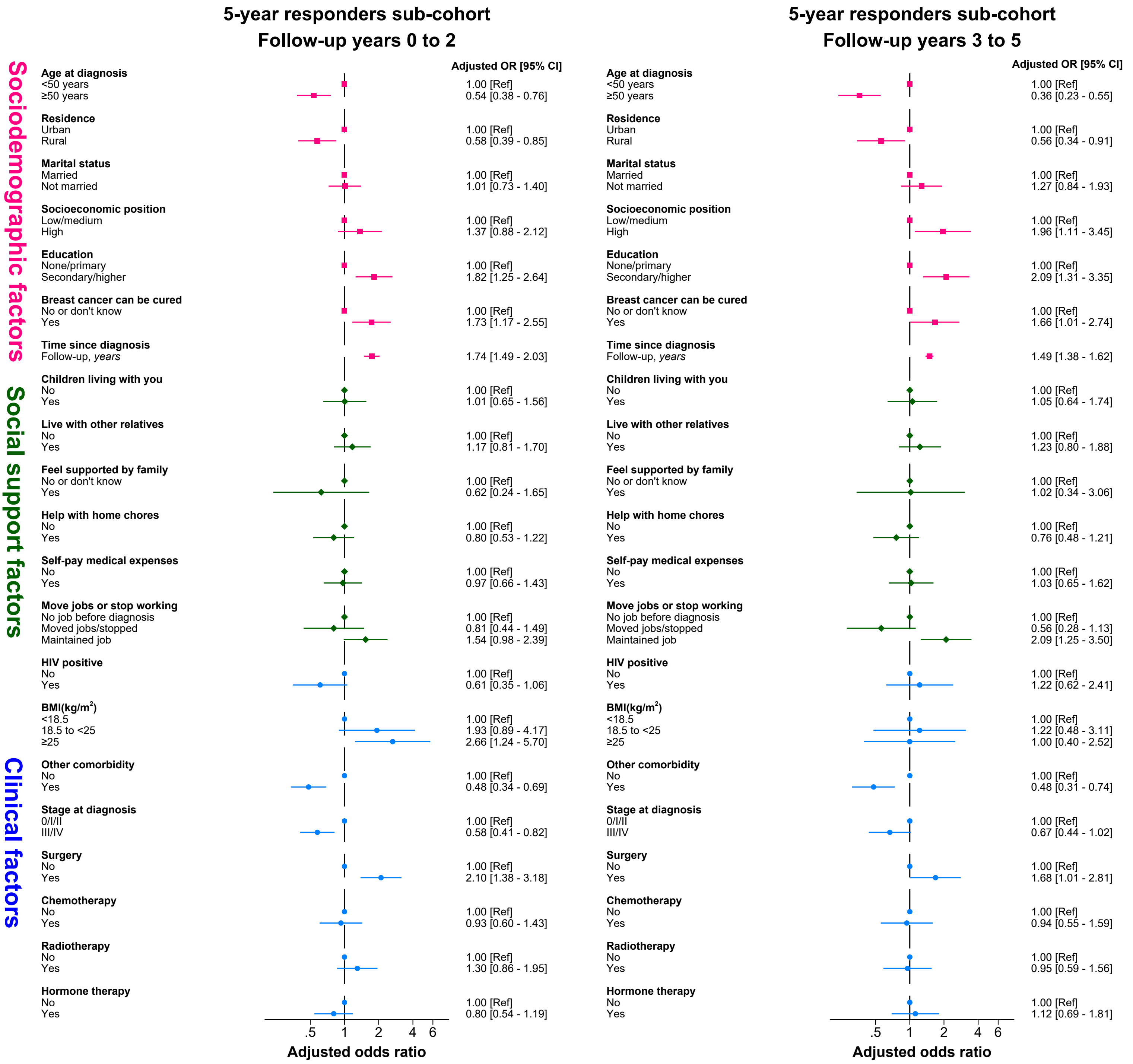

Supplementary Figure 6. Correlates of HRQoL, by time since diagnosis, in the 5-year responders subcohort of women diagnosed with breast cancer.

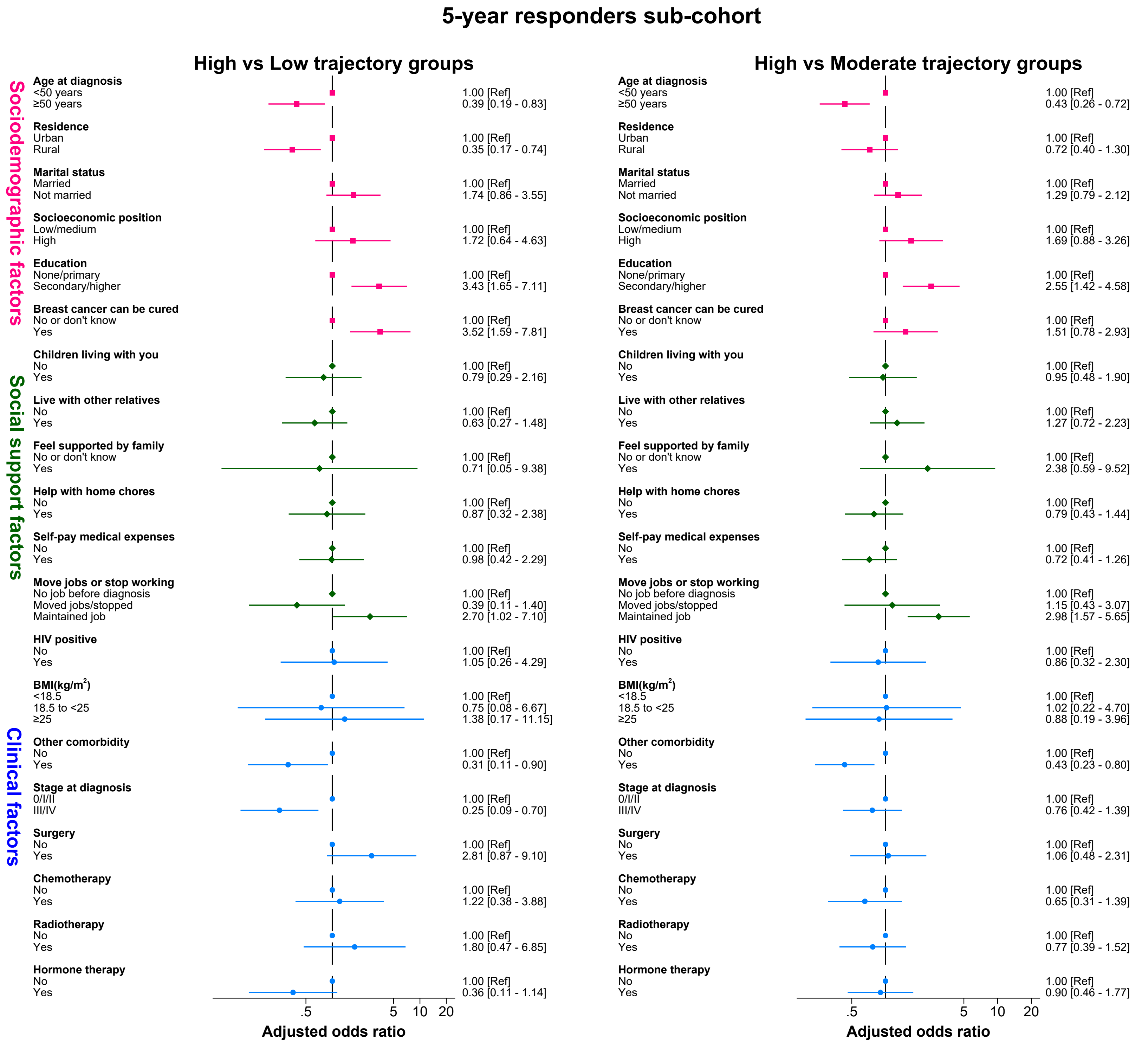

Supplementary Figure 7. Binary logistic regression analysis of the factors associated with HRQoL trajectory group membership in the 5-year responders sub-cohort of women diagnosed with breast cancer. Socioeconomic position, education, children living with you, BMI, and stage at diagnosis were re-grouped due to small numbers.
