# Supplementary material for: Longitudinal changes in health‐related quality of life after a breast cancer diagnosis in sub‐Saharan Africa: Evidence from the prospective ABC‐DO cohort
Source: Int J Cancer. 2026 Jan 29;159(1):78–91. doi: 10.1002/ijc.70350 (PMC13139992; doi:10.1002/ijc.70350)
Supplement: Supplementary file 2 — Table S1. Comparison of transformed EORTC QLQ‐C30 HRQoL scores. [file IJC-159-78-s002.pdf]

Supplementary Table 1. Comparison of transformed EORTC QLQ-C30 HRQoL scores

**Studies**	**EORTC QLQ-C30 HRQoL scores**			
	**Median (IQR)**			**Mean (SD)**
**Present study:**				
3 months after diagnosis	66.7 (33.3 - 83.3)			65.0 (24.0)
5 years after diagnosis	83.3 (66.7 - 100)			84.1 (18.2)
**EORTC reference scores**	66.7 (50.0 - 83.3)			61.8 (24.6)
**Global meta-analysis[a,1]:**	-			
Africa (including sub-Saharan Africa)	-			58.69 (0.89)
Global	-			64.72 (7.81)
**SSA studies, 2016 to 2025**	**Study design**	**Sample size**	**Time since breast cancer diagnosis at HRQoL assessment**	**Mean (SD) score**
Abegaz et al., 2018[2]	Cross-sectional	150	Mean=13.4 SD=12.1 months	52.7 (20.1)
Sibhat et., 2019[3]	Cross-sectional	404	<12 to ≥61 months (52.7% <12 months)	59.32 (22.94)
Hassen et al., 2019[4]	Cross-sectional	404	<12 to >48 months	52.98 (25.61)
Gadisa et al., 2019[5]	Longitudinal study[b]	146	NR	57.39 (20.1)
Aberaraw et al., 2020[6]	Cross-sectional	214	<12 to ≥60 months (72.5% <12 months)	83.61 (20.9)
Abebe 2020[7]	Cross-sectional	86	NR	48.25 (NR)
Odikpo et al., 2021[8]	Quasi-experimental	94	<3 to ≥10 months (44.7% ≥10 months)	40.0 (10.3)
Getu et al., 2022[9]	Cross-sectional	248	NR	65.6 (18.6)
Kidayi et al., 2023[10]	Cross-sectional	414	Mean=17.28 SD=21.98	63.4 (20.0)
Adam et al., 2023[11]	Cross-sectional	411	<12 to >60 months (57.4% <12 months)	61.3 (20.8)
Odo et al., 2024[12]	Cross-sectional	76	NR	69.3 (21.9)
Alem et al., 2024[13]	Cross-sectional	256	≤12 to >36 months (78.1% in ≤12)	70.6 (13.9)
Nsaful et al., 2024[14]	Cross-sectional	253	NR	Median: 83.3 (IQR=66.7-91.7)

NR: Not Reported; SSA: sub-Saharan Africa

In PubMed, we searched using the following search terms: "Quality of Life", "Health-related Quality of Life", "HRQOL", "QOL", "sub-Saharan Africa", "Breast cancer" for studies published in any language between 2015 and 2025. We included only studies that assessed HRQoL using the EORTC QLQ-C30.

[a] This meta-analysis included studies published between 2000 and 2021. The list of studies from SSA, or from anywhere else, that were included in the meta-analysis is not provided in the paper.

[b] Conducted between January and May 2017. Participants were interviewed twice on the first interview took place during the first cycle of chemotherapy and the second during the second cycle of chemotherapy.

References

1. Javan Biparva A, Raoofi S, Rafiei S, Pashazadeh Kan F, Kazerooni M, Bagheribayati F, Masoumi M, Doustmehraban M, Sanaei M, Zarabi F, Raoofi N, Beiramy Chomalu Z, Ahmadi B, Seyghalani Talab F, Sadat Hoseini B, Asadollahi E, Mir M, Deylami S, Zareei M, Sanaei H, Dousti Nia Kakavand F, Koohestani H, Nasiri M, Vali N, Ghashghaee A. Global quality of life in breast cancer: Systematic review and meta-analysis. BMJ Support Palliat Care. 2022;13(e3):E528–E536. PMID: 35710706

2. Abegaz TM, Ayele AA, Gebresillassie BM. Health Related Quality of Life of Cancer Patients in Ethiopia. J Oncol. 2018;2018.

3. Sibhat SG, Fenta TG, Sander B, Gebretekle GB. Health-related quality of life and its predictors among patients with breast cancer at Tikur Anbessa Specialized Hospital, Addis Ababa, Ethiopia. Health Qual Life Outcomes. Health and Quality of Life Outcomes; 2019;17(1):1–10. PMID: 31690327

4. Hassen AM, Taye G, Gizaw M, Hussien FM. Quality of life and associated factors among patients with breast cancer under chemotherapy at Tikur Anbessa specialized hospital, Addis Ababa, Ethiopia. PLoS One [Internet]. 2019;14(9):1–13. Available from: http://dx.doi.org/10.1371/journal.pone.0222629 PMID: 31539399

5. Gadisa DA, Gebremariam ET, Ali GY. Reliability and validity of Amharic version of EORTC QLQ-C30 and QLQ-BR23 modules for assessing health-related quality of life among breast cancer patients in Ethiopia. Health Qual Life Outcomes. Health and Quality of Life Outcomes; 2019;17(1):1–8. PMID: 31830992

6. Aberaraw R, Boka A, Teshome R, Yeshambel A. Social networks and quality of life among female breast cancer patients at Tikur Anbessa specialized hospital, Addis Ababa, Ethiopia 2019. BMC Womens Health. BMC Women's Health; 2020;20(1):1–9. PMID: 32160874

7. Abebe E, Demilie K, Lemmu B, Abebe K. Female Breast Cancer Patients, Mastectomy-Related Quality of Life: Experience from Ethiopia. Int J Breast Cancer. 2020;2020.

8. Odikpo LC, Chiejina EN. Assessment of Practice and Outcome of Exercise on Quality of Life of Women with Breast Cancer in Delta State. Asian Pac J Cancer Prev. 2021;22(8):2377–2383. PMID: 34452549

9. Getu MA, Chen C, Wang P, Kantelhardt EJ, Addissie A. Quality of life and its influencing factors among breast cancer patients at Tikur Anbessa specialised hospital, Addis Ababa, Ethiopia. BMC Cancer [Internet]. BioMed Central; 2022;22(1):1–12. Available from: https://doi.org/10.1186/s12885-022-09921-6 PMID: 35978281

10. Kidayi PL, Pakpour AH, Saboonchi F, Bray F, Manhica H, Mtuya CC, Serventi F, Aune RE, Mahande MJ, Björling G. Cross-Cultural Adaptation and Psychometric Properties of the Swahili Version of the European Organization for Research and Treatment of Cancer (EORTC) QLQ-BR45 among Breast Cancer Patients in

Tanzania. Healthc. 2023;11(18).

11. Adam R, Haileselassie W, Solomon N, Desalegn Y, Tigeneh W, Suga Y, Gebremedhin S. Nutritional status and quality of life among breast Cancer patients undergoing treatment in Addis Ababa, Ethiopia. BMC Womens Health. 2023;23(1):1–12. PMID: 37568125

12. Odo BA, Kouassi KKY, Kodjo W, Sessegnon FA, Toure PGL, Toure YL, Mebiala NMP, Nogbou ABY, Traore S, Yapo IN guessa. S blanc, Toure M, Adoubi I. Health-related quality of life and associated factors in breast cancer patients in Abidjan (Ivory Coast). J Cancer Policy. 2024;42(November).

13. Alem T, Nigatu D, Birara A, Fetene T, Giza M. Quality of life of breast cancer patients in Amhara region, Ethiopia: A cross-sectional study. PLoS One [Internet]. 2024;19(6 June):1–15. Available from: http://dx.doi.org/10.1371/journal.pone.0305263 PMID: 38935776

14. Nsaful J, Nartey ET, Dedey F, Bediako-Bowan A, Appiah-Danquah R, Darko K, Ankrah LNA, Akli-Nartey C, Annan JY, Dei-Asamoa J, Ahene-Amanquanor GA, Clegg-Lamptey JN. Quality of Life after Mastectomy with or without Breast Reconstruction and Breast-Conserving Surgery in Breast Cancer Survivors: A Cross-Sectional Study at a Tertiary Hospital in Ghana. Curr Oncol. 2024;31(6):2952–2962. PMID: 38920708
